# Edible Coatings Enriched with Essential Oils on Apples Impair the Survival of Bacterial Pathogens through a Simulated Gastrointestinal System

**DOI:** 10.3390/foods8020057

**Published:** 2019-02-04

**Authors:** Ana Isabel Vieira, Adriana Guerreiro, Maria Dulce Antunes, Maria da Graça Miguel, Maria Leonor Faleiro

**Affiliations:** 1 University of Algarve, FCT, Center for Biomedical Research, Edf. 8, Campus de Gambelas, 8005-139 Faro, Portugal; ana.isabelvieira@hotmail.com; 2 University of Algarve, FCT, Meditbio, Edf. 8, Campus de Gambelas, 8005-139 Faro, Portugal; adrianaguerreiro1@gmail.com (A.G.); mantunes@ualg.pt (M.D.A); mgmiguel@ualg.pt (M.d.G.M.)

**Keywords:** apples, edible coatings, foodborne pathogens

## Abstract

Edible coatings supplemented with essential oil components have been investigated to control spoilage microorganisms. In this study, the survival of *Listeria monocytogenes* and *Salmonella enterica* serovar Typhimurium on apples treated with edible coatings based on sodium alginate (2%) (ECs) and supplemented with essential oil components, namely eugenol (Eug) at 0.2% or in combination with 0.1% (v/v) of Eug and citral (Cit) at 0.15% was determined. Both bacterial pathogens were exposed on apples treated with ECs supplemented with Eug or Eug + Cit and challenged with gastrointestinal fluids and their survival was examined. Both pathogens were able to survive on the surface of ‘Bravo de Esmolfe’ apple. The use of ECs in fresh-cut fruits impaired the survival of both bacterial populations over 72 h at 4 °C. The exposure of the pathogens on apples with ECs supplemented with Eug and Cit and challenged with gastrointestinal fluids significantly reduced their survival. This study evidences that the use of alginate edible coating enriched with Eug or the combination of Eug and Cit can contribute to the safer consumption of minimally processed fruits.

## 1. Introduction

Foodborne outbreaks associated with the consumption of fruits are not frequent; however, their numbers have been increasing worldwide [1,2]. A listeriosis outbreak linked to the consumption of caramel apples (produced with ‘Granny Smith’ and ‘Gala’ apples) was reported in 12 states of USA with a total of 35 people infected, 34 hospitalizations, and seven deaths) [3]. Changes in diet including higher consumption of fruits and vegetables is highly recommended to decrease the risk of several diseases, such as coronary heart disease, cardiovascular disease, stroke, total cancer, and hypertension [4,5,6]. The elderly, pregnant women, and immunocompromised patients are at risk of listeriosis [7]; for this risk group the provision of safe fruit and vegetables is critical. In minimally processed produce microbial contamination may occur at several production steps, for example, at orchards, harvest, storage, processing, and distribution [8,9]. Such contamination may be reinforced by abusive storage temperature, humidity, injured location, nutrients richness (as sugars), and pH value. The consumption of apple cider contaminated with *Escherichia coli* O157:H7 and *Cryptosporidium* resulted in outbreaks [10,11] and several product recalls were noticed due to possible *E. coli* O157:H7 contamination [12]. Besides apples and their food derivatives being characterized by a low pH value (3–4) that can inhibit the bacterial growth, many foodborne pathogens are able to overcome such low pH by displaying stress tolerance responses, challenging food control measures [13]. Fresh-cut fruit is consumed without further microbial control procedures, so effective measures that ensure product safety are necessary, especially because in these products cross-contamination during the peeling and cutting operations may occur. The processing of the fresh-cut fruits includes key preservation techniques, namely the use of packaging with modified atmosphere (modified atmosphere packing) that can be improved with the addition of antimicrobial and antioxidant agents allowing, in combination with low storage temperature, an extended shelf life, at the same time preserving their nutritional and organoleptic proprieties [14,15]. The use of edible coatings supplemented with essential oils can constitute a promising approach to improve both preservation and safety of fresh-cut fruits maintaining the use of the conventional packing technologies [14]. The survival of foodborne pathogenic bacteria, such as *Salmonella* spp. and *Listeria monocytogenes*, in fresh-cut apples has been reported, representing a public health problem [16,17]. The incorporation of essential oils into edible coatings used in fresh-cut apples to control *L. innocua* was reported [18]. However, the composition of the essential oils may significantly vary over the years, so the use of essential oil components into edible coatings will ensure steady properties, meeting the market needs [14,19,20]. Eugenol and citral are two essential oil components that are used as flavoring agents in food products. The European Commission approved their use in the food industry (Regulation EU 872/2012). The incorporation of these components into chitosan edible coatings not only reduced the microbial decay, but also conserved the nutritional and sensory quality of *Arbutus unedo* fruits [20]. In another study, the use of alginate edible coating supplemented with eugenol or a combination of eugenol and citral achieved similar results on the preservation of fresh-cut ‘Bravo de Esmolfe’ apple, a Portuguese apple cultivar [21]. The consumption of ‘Bravo de Esmolfe’ apple is increasing due to its agreeable characteristics (flavor, sweetness, and a particular aroma); however, during storage these proprieties significantly decline, becoming more susceptible to cross-contamination [21,22]. The use of edible coatings can not only assure the preservation of the nutritional quality and avoid the microbial spoilage, but also limit the colonization of the fruit by foodborne pathogens.

The aim of this study was to evaluate the effect of edible coatings supplemented with essential oil components on the survival of *Listeria monocytogenes* and *Salmonella enterica* on apples and estimate the impact of the edible coatings on the survival of the foodborne pathogens during exposure to a simulated gastrointestinal system.

## 2. Materials and Methods 

### 2.1. Bacterial Strains

The tested strains were *Listeria monocytogenes* 12.04 (serovar 1/2b) a salad isolate (a gift from the Centre National de Référence des Listeria, Institut Pasteur, France (CLIP 88411)) and *Salmonella enterica* subsp. *enterica* serovar Typhimurium ATCC 14028. The bacterial strains were recovered from storage at −80 °C in Tryptic Soy Agar (TSA) and maintained at 4 °C. Preceding each assay, the bacterial cells were transferred to fresh TSA plates and incubated at 30 °C during 18–24 h. The pre-inoculum culture was prepared by transferring a loopful of the previous TSA plate into 10 mL of tryptic soy broth (TSB), which was incubated overnight at 30 °C in a shaking water-bath at 120 rpm.

### 2.2. Essential Oils Components

The essential oil components eugenol (99%) and citral (95%) were purchased from Sigma Aldrich (Steinheim, Germany).

### 2.3. Fruit

In this study two apple cultivars were used, the *Malus domestica* Borkh. cv. Bravo de Esmolfe, a Portuguese apple cultivar produced in northern Portugal, and the commercial cv. Golden Delicious. Fruits of cv. Bravo de Esmolfe and Golden Delicious were purchased in the local market at the eating-ripe stage, in conditions to be processed as fresh-cut. The fruits were selected for uniformity of size and absence of flaws. 

### 2.4. Organic Acid Content

The organic acid composition and quantification were determined by high-performance liquid chromatography (HPLC) coupled with a diode array detector (DAD). Five grams of the pulp was collected from five fruits and the samples were freeze-dried. The freeze-dried samples were maintained at −80 °C until their analysis. An aliquot of 50 ± 0.5 mg was diluted in 3 ml of Milli-Q water. The mixture was vigorously mixed in a vortex followed by 5 min rest [23,24]. After that the mixture was filtered (0.2 μM), having been previously stirred. The collected filtrate was analyzed on a HPLC binary pump system equipped with a diode array detector (DAD, L-2455, Elite LaChrom series, Hitachi, Japan) with a multiple wavelength detector, degasser, and cooled autosampler. The filtered sample extract (20 µL) was injected into a Purospher Star RP-18 column (4.6 mm diameter × 250 mm, 5 µm particle size; Merck Millipore, Germany) with an organic acid guard column (LiChroCART 4-4 Merck Millipore, Germany). The temperature of the column was set to 35 °C using a thermostated column compartment (L-2300, Elite LaChrom series, Hitachi, Japan). The mobile phase used was 0.2% HPLC-grade aqueous metaphosphoric acid at a flow rate of 1.0 mL/min. Non-volatile organic acids were detected at 210 nm and quantified using linear standard curves (0.01–2.5 mg/mL; average R2 = 0.99).

### 2.5. Determination of pH

Five fruits without peel and seeds were sliced and ground. The ground fruits were filtered through a gauze tissue, collecting the juice. The pH was determined using the collected juice (GLP 21 Crison Instruments, Barcelona, Spain). The determinations were done in triplicate.

### 2.6. Edible Coatings

The formulation of the edible coatings based on sodium alginate (2%) was done as previously described [20,25]. The edible coatings were supplemented with eugenol (Eug) or a combination of citral (Cit) and Eug. The concentrations of the essential oil components were selected based on previously determined minimum inhibitory concentrations (MIC) for Eug and Cit [20]. The tested edible coatings were 1) sodium alginate (2%) with 0.2% Eug, 2) sodium alginate (2%) with 0.1% (v/v) of Eug, and 0.15% of Cit. For the application of the edible coatings, the fruits were previously disinfected with 70% ethanol and dried in a laminar flow chamber. Afterward, each fruit was sliced and the slices were transferred to sterile Petri dishes. Each slice was inoculated with 10 μL (10^9^ CFU/mL) of the previously prepared bacterial culture in an area of 1 × 1 cm. After that, each slice was firstly immersed in a solution of ascorbic acid (1%) (Fisher Chemical) for 1 min to avoid browning and then transferred to the edible coating solution for 2 min and finally dipped in a CaCl_2_ (1%) solution (Panreac) during 1 min for cross-linking. The fruits were then maintained at 4 °C. Fruits with no edible coating were used as a control. The experiment was done in triplicate. The viability was determined as described above.

### 2.7. Survival of Bacteria on the Surface of Apples

The bacterial culture was prepared by inoculating 9 mL of tryptic soy broth (TSB) with 500–1000 μL of the previous pre-inoculum culture and allowed to grow until an optical density 600 nm reached 0.3–0.4 (10^8^–10^9^ CFU/mL).

In a laminar flow cabinet, the surface of the fruits was disinfected with 70% ethanol and left to dry. Following this, the fruits were manually cut in two and each half was placed in a sterile Petri dish with the cut surface down. At the top of an area of 3 × 3 cm of the fruit, 100 microliters of the bacterial culture were distributed. The inoculum was dried for 30 min. The fruits were incubated at 20 °C.

The viability of the bacterial cells was determined by preparing decimal dilutions of the homogenate of 10 g of the fruit with 90 mL of peptone water (Oxoid, Basingstoke, Hampshire, UK). The decimal dilutions were plated on TSA plates. The experiments and the viability determinations were done in triplicate.

### 2.8. Exposure to Simulated Gastrointestinal Fluids

The components of gastrointestinal fluids were purchased from Sigma-Aldrich (Madrid, Spain) and VWR (Lisbon, Portugal).

The impact of the edible coatings on the survival of the bacterial cells exposed to simulated gastrointestinal fluids was evaluated using the edible coating sodium alginate (2%) with 0.2% Eug and the sodium alginate (2%) with 0.1% (v/v) of Eug and 0.15% (v/v) of Cit. The experiment was done with the cv ‘Bravo de Esmolfe’. Fruits with no edible coating were used as a control. The application of the edible coatings was done as described above. Before the challenge with the simulated gastrointestinal juices, the fruits were maintained at 4 °C for 24 h to reproduce a more realistic commercial condition. 

The exposure of inoculated and edible-coated fruits to simulated gastrointestinal fluids was done as previously described [26,27]. Ten grams of fruit was transferred to a sterile Stomacher bag (Interscience, Saint Nom, France) and 6 ml of artificial saliva (KCl (89.6 g/L); KSCN (20 g/L); NaH2PO4 (88.8 g/L); Na2SO4 (57 g/L); NaCl (175.3 g/L); NaHCO3 (84.7 g/L); urea (25 g/L); 290 mg of α-amylase; 15 mg of uric acid; 25 mg mucin, pH 6.8 ± 0.2) was added. The fruit was homogenized for 30 sec in a Stomacher (IUL Instruments, Barcelona, Spain) and the incubation was done for 5 min, 37 °C, 60 rpm. After this time interval sampling was done to determine the viability as described above, and an artificial gastric fluid (1.884 mL NaCl (175.3 g/L); 0.36 mL NaH2PO4 (88.8 g/L); 1.104 mL KCl (89.6 g/L); 2.16 mL CaCl_2_·2H_2_O (22.2 g/L); 1.2 mL NH_4_Cl (30.6 g/L); 0.09 mL HCl 37% (w/w); 1.2 mL glucose (65 g/L); 1.2 mL glucuronic acid (2 g/L); 0.408 mL urea (25 g/L); 1.2 mL glucosamine hydrochloride (33 g/L); 0.12 g bovine serum albumin; 0.3 g pepsin; 30 mL mucin (12 g/L), pH 2.5 or pH 1.5) was added to the previous saliva mixture. Incubation continued for 2 h, at 37 °C and a slight agitation (60 rpm). Simulation of the gut environment was achieved by the addition of an artificial intestinal fluid (pH 6.5) to the gastric medium. The intestinal fluid composition includes 2.4 mL NaCl (175.3 g/L); 2.4 mL NaHCO_3_ (84.7 g/L); 0.6 mL KH_2_PO_4_ (8 g/L); 0.378 mL KCl (89.6 g/L); 0.6 mL MgCl_2_ (5 g/L); 0.0108 mL HCl 37% (w/w); 0.24 mL urea (25 g/L); 0.54 mL CaCl_2_·2H_2_O (22.2 g/L); 0.06 g BSA; 0.54 g pancreatin; 0.09 g lipase and bile solution is made of 1.2 mL NaCl (175.3 g/L); NaHCO_3_ (84.7 g/L); 0.168 mL KCl (89.6 g/L); 0.006 mL HCl 37% (w/w); 0.4 mL urea (25 g/L); 0.4 mL CaCl_2_·2H_2_O (22.2 g/L); 0.072 g BSA; 1.2 g bile (0.6 g porcine bile, 0.6 g bovine bile). The intestinal challenge lasted 2 h at 37 °C, 60 rpm. Three independent experiments were performed. The viability in the gastric and intestinal challenge was determined after 1 and 2 h of exposure.

### 2.9. Statistical Analyses

The statistical differences were determined by one-way ANOVA using the SPSS 22.0 programme. Differences were considered statistically significant at *p* < 0.05.

## 3. Results

### 3.1. Organic Acids, Sugar, and pH 

The pH value and the main identified organic acids and sugars in the tested apple fruits are summarized in Table 1. The lowest pH value was observed in the ’Bravo de Esmolfe’ apples (4.25 ± 0.06), in contrast, the ’Golden Delicious’ apples showed a higher pH value (4.97 ± 0.04). The major identified organic acids were oxalic, malic, and quinic acid, and no significant differences (*p* > 0.05) were observed in the acid content between the two cultivars (Table 1). Fructose was the main sugar in both cultivars, but the cv ’Bravo de Esmolfe’ showed the highest level (*p* < 0.05) (17.61 ± 1.13 g/100 g of dry weight) (Table 1).

### 3.2. Survival of Foodborne Pathogens on the Surface of ‘Bravo de Esmolfe’ Apples

The ability of the two foodborne pathogens to survive on the surface of the apples was estimated using the cv “Bravo de Esmolfe”. The survival of *L. monocytogenes* 12.04 and *Salm*. Typhimurium ATCC 14028 on the surface of ’Bravo de Esmolfe’ apples is shown in Table 2. *L. monocytogenes* 12.04 showed a higher ability to survive on the surface of the fruits in comparison to *Salm*. Typhimurium ATCC 14028. The percentage of viable cells of *L. monocytogenes* 12.04 after the first day of storage at 20 °C was 81.2 ± 10.30%, in contrast, the viability of *Salm*. Typhimurium ATCC 14028, in the same time interval, was 56.38 ± 8.90%. After seven days of storage, the difference between the survival of the two foodborne pathogens was even more pronounced, 73.64 ± 3.48% of *L. monocytogenes* 12.04 cells were viable, in comparison to *Salm*. Typhimurium ATCC 14028 for which no cells were recovered after the same period of time.

### 3.3. The Effect of Edible Coatings on the Survival of the Foodborne Pathogens in Fresh-Cut Apples

The results of the effect of edible coatings on the survival of *L. monocytogenes* and *Salm*. Typhimurium on the fresh-cut fruits are summarized in Table 3. The survival of both foodborne pathogens in apples with no edible coating (control) was significantly lower (*p* < 0.05) in ’Bravo de Esmolfe‘ than in ‘Golden Delicious‘ fresh-cut apples stored at 4 °C for 72 h (Table 3). In ’Bravo de Esmolfe’ fresh-cut apples stored at 4 °C after 24 h, *L. monocytogenes* and *Salm.* Typhimurium survived significantly less (*p* < 0.05) in fruits treated with any edible coating tested in comparison to the control (Table 3). However, after 72 h, *L. monocytogenes* survived significantly less (*p* < 0.05) in fruits treated with the edible coating supplemented with the combination of 0.1% Eug and 0.15% Cit (Table 3). In contrast, *Salm*. Typhimurium, after the same time interval, survived similarly (*p* > 0.05), regardless of the edible coating tested, but significantly less (*p* < 0.05) than in the control (Table 3).

In ’Golden Delicious’ fresh-cut apples, stored at 4 °C after 24 h, *L. monocytogenes* survived significantly less in fruits with edible coating supplemented 0.2% Eug, but *Salm*. Typhimurium showed a tendency to be more susceptible to the edible coating with the combination of 0.1% Eug and 0.15% Cit. Although no statistically significant differences were found at 72 h, *L. monocytogenes* showed to be more susceptible to both tested edible coatings, in contrast, to *Salm*. Typhimurium that showed a similar survival (*p* > 0.05) either in edible coating apples or control fruit (Table 3). 

### 3.4. The Impact of Edible Coatings on the Ability of Foodborne Pathogens to Overcome a Simulated Gastrointestinal Challenge 

The survival of the foodborne pathogens in apples with the edible coatings challenged with artificial gastrointestinal fluids was performed using the fruits of the cv “Bravo de Esmolfe”. The challenge with the simulated gastrointestinal fluids was done determining the survival of two foodborne pathogens with gastric fluid at two pH values, pH 2.5 and pH 1.5. The exposure of the foodborne pathogens in apples with edible coatings to gastrointestinal fluids was done after 24 h at 4 °C reproducing a possible commercial condition.

**Figure 1:**
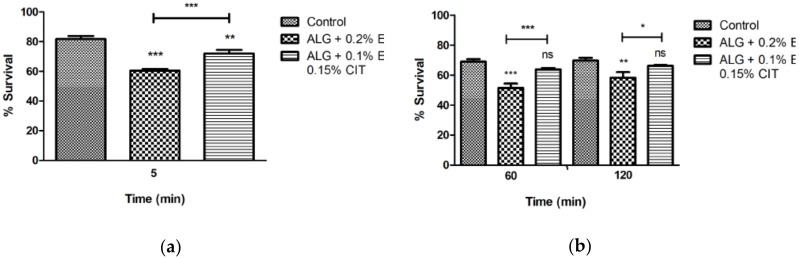

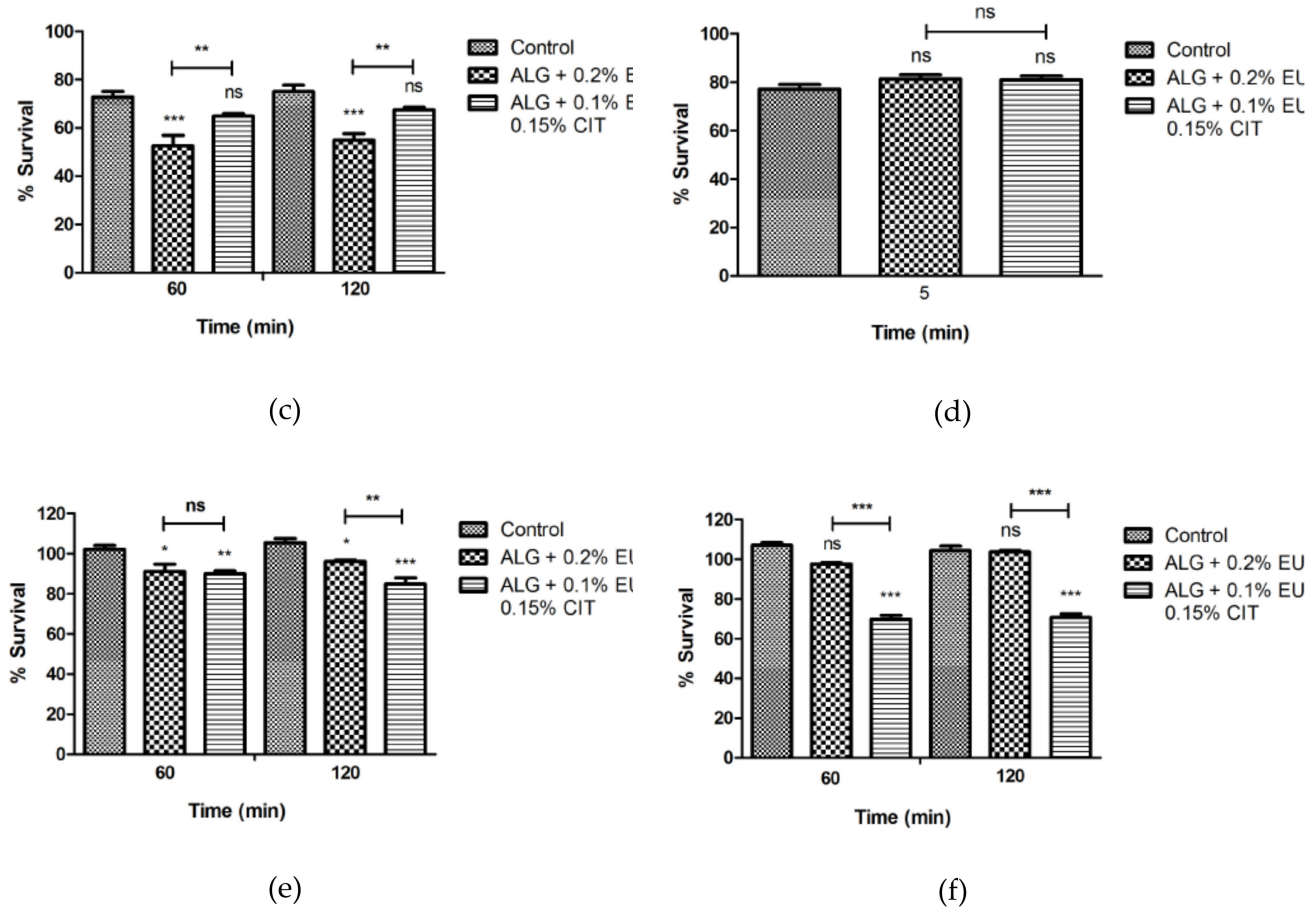
Survival of *Listeria monocytogenes* 12.04 (**A**–**C**) and *Salmonella* enterica serovar Typhimurium (**D**–**F**) inoculated in edible coating apples (apples prior to the gastrointestinal challenge were maintained at 4 °C for 24 h) during exposure to simulated gastrointestinal system (**A**, **D**-saliva; **B**, **E**-gastric fluid (pH 2.5); **C**, **F**-intestinal fluid and bile). Data are the mean of three independent experiments. Error bars represent the standard deviation. Significant survival differences *p < 0.05, ** *p* < 0.01, *** *p* < 0.001, ns, not significant. The bars show significant differences between the edible coatings. ALG-Alginate, EUG-Eugenol, CIT-Citral.

**Figure 2:**
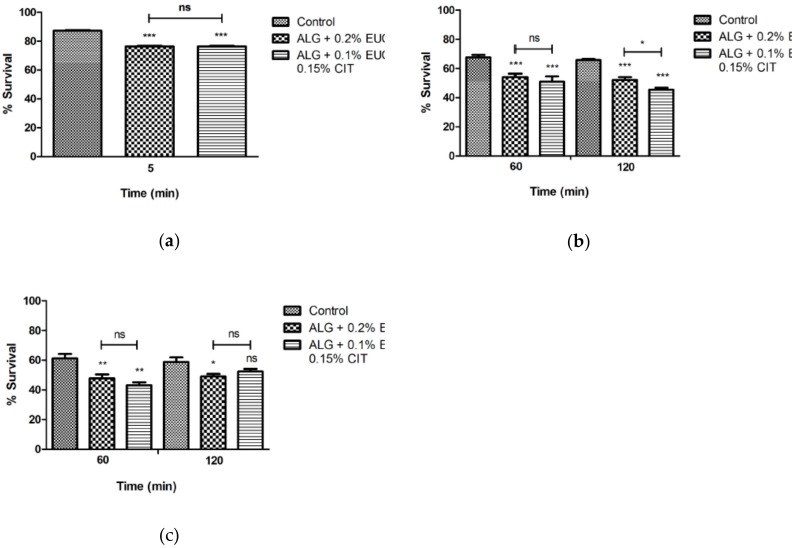
Survival of *Salmonella* enterica serovar Typhimurium (**A**–**C**) inoculated in edible coating apples (apples prior to the gastrointestinal challenge were maintained at 4 °C for 24 h) during exposure to simulated gastrointestinal system (**A**-saliva; **B**-gastric fluid (pH 1.5); **C**-intestinal fluid and bile). Data are the mean of three independent experiments. Error bars represent the standard deviation. Significant survival differences * *p* < 0.05, ** *p* < 0.01, *** *p* < 0.001, ns, not significant. The bars show significant differences between the edible coatings. ALG-Alginate, EUG-Eugenol, CIT-Citral.

The results of the survival of *L. monocytogenes* and *Salm.* Typhimurium in apples with edible coatings and challenged with gastrointestinal fluids are illustrated in Figure 1 and Figure 2.

The exposure to saliva caused a significant reduction (*p* < 0.05) of *L. monocytogenes* survival when both edible coatings were used (Figure 1A). In contrast, the exposure to saliva did not cause any reduction of *Salm*. Typhimurium survival whatever the edible coating used, in comparison to the control (Figure 1D). The survival of *L. monocytogenes* during the exposure to the gastric medium (the added gastric fluid had a pH 2.5 and the final pH of the mixture of the macerated apple plus the gastrointestinal fluids was for the control 5.69 ± 0.15, for ALG + 0.2% Eug was 5.49 ± 0. 36 and for ALG + 0.1% Eug and 0.15% Cit was 5.46 ± 0.05) was significantly lower (*p* < 0.05) when the edible coating supplemented with 0.2% Eug was used, either after 1 or 2 h (Figure 1B). A different behavior was observed in the survival of *Salm.* Typhimurium, which was more affected after 2 h exposure (*p* < 0.05) when the edible coating with 0.1% Eug and 0.15% Cit was used (Figure 1E). The simulation of the gut environment by the addition of the artificial intestinal fluid caused the highest reduction of the survival of *L. monocytogenes* when the edible coating with 0.2% Eug was applied (Figure 1C). Differently, the survival of *Salm*. Typhimurium was more affected in the presence of the edible coating supplemented with 0.1% Eug and 0.15% Cit (Figure 1F).

The experiment using a lower pH of the gastric fluid (pH 1.5) showed the highest susceptibility of *L. monocytogenes* exposed to both edible coatings as no cells were recovered after the challenge with the gastrointestinal fluids, in contrast, to the control cells that were able to survive. After 2 h of exposure to the gastric juice 55.07 ± 7.82% of *L. monocytogenes* cells from the control apples were recovered, and after 2 h of exposure to the simulated intestinal medium the viability achieved 50.95 ± 5.06%. In contrast, *Salm.* Typhimurium showed to be more resistant to the gastrointestinal challenge. Nevertheless, the use of edible coating supplemented with essential oil components impaired the survival of *Salm.* Typhimurium during the gastrointestinal challenge (Figure 2A–C). After 2 h of exposure to gastric fluid the survival of *Salm.* Typhimurium was significantly lower (*p* < 0.05) when 0.1% Eug and 0.15% Cit was used (Figure 2B). After the addition of the simulated intestinal medium the survival of *Salm.* Typhimurium was equally lower in the presence of both edible coatings in comparison to the control cells (Figure 2C).

## 4. Discussion

Minimally processed fruits are susceptible to contamination, and although foodborne outbreaks linked with the consumption of fruits are rare, recently their numbers are increasing. Techniques that can not only contribute to improving the shelf-life of fresh-cut fruit but also control foodborne pathogens, which are in general more resistant to frequent food preservative techniques are required.

Apples are characterized by a significant diversity of organic acids in their composition, with malic acid being the main organic acid [28,29]. The two apple cultivars showed similar levels of oxalic, malic, and quinic organic acids. However, the pH value was different between the two cultivars. Nevertheless, it is important to highlight that the pH value can vary according to the conditions of plant growth, and it is expected that some variation on this parameter can be found, for instances in the study of Alegre et al. [16] ’Golden Delicious’ apples showed a pH value of 4.16, a lower pH than the one found in our study. Fructose was the main sugar found in both cultivars. This is in agreement with other reported studies [28]. 

*Listeria monocytogenes* 12.04 showed a better fitness to survive on the surface of ’Bravo de Esmolfe’ apples in comparison to *Salm*. Typhimurium ATCC 14028. The ability of different foodborne pathogens to survive on the surface of whole and unblemished apples has been evaluated [17,30]. In the study of Perez-Rodriguez et al. [17], *Salm*. Typhimurium was able to survive 12 days on the surface of apples of the variety Summerred (that are grown in Norway) stored at 22 °C and 70% RH. In the study of Tian et al. [30], three strains of *Salm.* Typhimurium were also able to show high survival ability on the surface of ‘Golden Delicious’ apples, though the *Salm.* Typhimurium population was just monitored for two days at 15 °C. Our results and others show that once the apple surface became contaminated the persistence of both pathogens on the fruit surface may compromise their safe consumption and also their following processing.

The use of edible coatings with incorporated essential oils has shown to be an efficient method to extend the shelf life of different fruits [20,25,31]. The use of alginate edible coating enriched with eugenol or a combination of eugenol and citral has been previously demonstrated to efficiently preserve fresh-cut ‘Bravo de Esmolfe’ apple with a good sensory appreciation [21]. In our study, we show that the use of alginate edible coating supplemented with eugenol or a combination of eugenol and citral not only can improve the shelf life of apples but also impair the survival of two important foodborne pathogens improving the safety of these fruits. Interesting fresh-cut apples of the cv ‘Bravo de Esmolfe’ endured a lower survival of both *L. monocytogenes* and *Salm*. Typhimurium, in comparison to the cv ‘Golden Delicious’. This difference between the two cultivars may be related with the lower pH of the ‘Bravo de Esmolfe’ apples but also to components that were not in the present study investigated, such as the presence of phenols and terpenoids in the composition of this cultivar [32]. Such compounds including organic acids found in fruits are also known for their antimicrobial activity [13,33]. Eugenol causes injury to the cellular membrane of either Gram positive or Gram negative bacteria and disrupts the glucose metabolism [34]. Citral targets both the cytoplasmic and the outer membrane of *E. coli* cells [35], induces the formation of reactive oxygen species (ROS) [36], and alters the fatty acid composition of the membrane of several bacteria, including *L. monocytogenes* [37]. On the other hand, organic acids present in fruits can also act against bacterial cells, such activity has been associated with their lipid permeability and the consequent discharge of protons. Moreover, smaller the undissociated molecules of organic acids can more easily cross the bacterial membrane affecting a series of vital events, such as disturbing the intracellular pH, the membrane function, increasing the osmolarity to fatal turgor pressure, and finally disrupting essential metabolic pathways [13]. The antimicrobial activity of these organic acids, in particular, malic acid has been explored as a preservative in fresh producing in apples [38]. 

The use of edible coatings incorporating essential oil components can affect the survival of the two foodborne pathogens during their transition from apples to the host gastrointestinal tract. The impact of edible coatings supplemented with 0.2% Eug or the combination of 0.1% Eug + 0.15% Cit on the survival of *L. monocytogenes* and *Salm.* Typhimurium during the challenge with the simulated gastrointestinal system evidenced that the ability of the two foodborne pathogens to defeat the low pH of the stomach and the intestinal milieu was decreased, in comparison to the control cells. This impact was more pronounced when the bacterial cells were exposed to the gastric fluid at pH 1.5 even leading to no recovery of *L. monocytogenes* cells from samples containing the edible coatings supplemented with the essential oil components. At this lethal pH *Salm*. Typhimurium was shown to be more resistant, surviving the gastrointestinal challenge in comparison to *L. monocytogenes*. However, the survival of the *Salm*. Typhimurium cells from samples contained the edible coatings incorporated with the essential oil components was lower in comparison to control cells. The reports about the ability of either *L. monocytogenes* or *Salm.* Typhimurium to beat the gastrointestinal system using a food matrix are limited [27,39]. However, it is evident that these two foodborne pathogens are able to defeat the gastrointestinal barrier of the host [27,39,40]. The present study shows that the use of edible coatings enriched with eugenol and the combination of eugenol and citral causes an increase in the susceptibility of the two pathogens that ultimately impairs their survival during the transition from apples to the gastrointestinal tract. 

## 5. Conclusions

The results of this study show that *L. monocytogenes* and *Salm.* Typhimurium may persist on the surface of the cv ’Bravo de Esmolfe’ for several days, compromising their safe consumption and the following processing. The use of edible coatings enriched with Eug or the combination of Eug and Cit are useful tools to impair the survival of both pathogens on fresh-cut apples. In addition, our study shows for the first time, to the best of our knowledge, that their use increases the susceptibility of the pathogens in a manner that reduces their capacity to overcome the gastrointestinal stress. Altogether these results evidence that edible coatings enriched with essential oil components can significantly contribute to a safer consumption of minimally processed fruits.

## Figures and Tables

**Table 1:** Organic acid composition (g/100 g of dry weight), sugars (g/100 g of dry weight) and pH value.

Fruit	Oxalic Acid	Malic Acid	Quinic Acid	Fructose	Glucose	Sucrose	pH
“Bravo de Esmolfe”	1.87 ± 0.42 ^a^	1.71± 0.26 ^a^	1.16 ± 0.22 ^a^	17.61±1.13 ^a^	6.14±0.36 ^a^	4.93±1.25 ^a^	4.25 ± 0.06 ^b^
“Golden Delicious”	1.57 ± 0.21 ^a^	1.25 ± 0.36 ^a^	0.88 ± 0.22 ^a^	15.71±0.02 ^b^	6.81±0.30 ^a^	3.67±0.05 ^a^	4.97 ± 0.04 ^a^

Data represent the mean ± standard deviation of three determinations. Data in each column followed by the same letter are not significantly different (*p* > 0.05).

**Table 2:** Survival of *L. monocytogenes* 12.04 and *S. Typhimurium* ATCC 14028 on the surface of “Bravo de Esmolfe” apples stored at 20 °C for 7 days.

Time (Days)	Survival (%)	
	*L. monocytogenes* 12.04	*Salm.* Typhimurium ATCC 14028
0	100.00 ± 0.00 ^a^	100.00 ± 0.00 ^a^
1	81.27 ± 10.30 ^b^	56.38 ± 8.90 ^b^
2	75.73 ± 4.87 ^b^	46.76 ± 5.11 ^c^
3	74.02 ± 5.34 ^b^	54.74 ± 6.07 ^b^
7	73.64 ± 3.48 ^b^	NR

Data are the mean of three experiments ± standard deviation. Data in the same column followed by different letters are significantly different (*p* < 0.05). NR- Not recovered.

**Table 3:** The impact of edible coatings on the survival (%) of *L. monocytogenes* 12.04 and *Salm*. Typhimurium ATCC 14028 on fresh-cut apples stored at 4 °C.

Fruit	Time (h)	*L. monocytogenes* 12.04 (% Survival)			*Salm*. Typhimurium ATCC 14028 (% Survival)		
		Control	ALG (2%) with 0.2% EUG	ALG (2%) with 0.1% EUG and 0.15 % CIT	Control	ALG (2%) with 0.2% EUG	ALG (2%) with 0.1% EUG and 0.15 % CIT
‘Bravo de Esmolfe’	0	100.0 ± 0.0 ^aA^	100.0 ± 0.0 ^aA^	100.0 ± 0.0 ^aA^	100.0 ± 0.0 ^aA^	100.0 ± 0.0 ^aA^	100.0 ± 0.0 ^aA^
	24	62.50 ± 2.34 ^aD^	56.24 ± 8.28 ^bC^	57.94 ± 2.63 ^b,cD^	75.83 ± 12.78 ^aB^	56.56 ± 3.43 ^bC^	55.64 ± 5.63 ^bC^
	48	60.68 ± 5.21 ^aD^	58.56 ± 8.19 ^bC^	59.72 ± 3.93 ^bD^	61.59 ± 4.13 ^aC^	52.36 ± 3.83 ^bC^	50.94 ± 3.64 ^bC^
	72	62.58 ± 4.56 ^aD^	58.37 ± 3.31 ^bC^	55.55 ± 3.75 ^cD^	60.48 ± 5.67 ^aC^	55.60 ± 4.89 ^bC^	52.70 ± 5.80 ^bC^
‘Golden Delicious’	0	100.0 ± 0.0 ^aA^	100.0 ± 0.00 ^aA^	100.0 ± 0.0 ^aA^	100.0 ± 0.0 ^aA^	100.0 ± 0.0 ^aA^	100.0 ± 0.0 ^aA^
	24	72.17 ± 5.89 ^aB,C^	67.38 ± 4.78 ^bB^	70.74 ± 5.45 ^aB,C^	75.13 ± 4.94 ^aB^	67.45 ± 5.35 ^b,cB^	66.68 ± 8.03 ^cB^
	48	70.55 ± 5.57 ^aC^	67.38 ± 7.21 ^aB^	67.77 ± 5.38 ^aC^	71.15 ± 7.62 ^aB^	65.13 ± 6.84 ^bB^	63.86 ± 4.58 ^bB^
	72	76.07 ± 4.48 ^aB^	74.52 ± 6.44 ^a,bB^	73.67 ± 4.94 ^a,bB^	72.85 ± 4.40 ^aB^	68.54 ± 3.96 ^aB^	67.21 ± 9.40 ^aB^

Data are the mean of three experiments ± standard deviation. For each cultivar and bacteria data in each raw with the same lower letter are not significantly different (*p* > 0.05). In each column data with a different capital letter are significantly different (*p* < 0.05). ALG-Alginate, EUG-Eugenol, CIT-Citral.
